# What is special about the human arcuate fasciculus? Lateralization, projections, and expansion

**DOI:** 10.1016/j.cortex.2018.05.005

**Published:** 2019-09

**Authors:** Nicole Eichert, Lennart Verhagen, Davide Folloni, Saad Jbabdi, Alexandre A. Khrapitchev, Nicola R. Sibson, Dante Mantini, Jerome Sallet, Rogier B. Mars

**Affiliations:** a Wellcome Centre for Integrative Neuroimaging, Centre for Functional MRI of the Brain (FMRIB), Nuffield Department of Clinical Neurosciences, John Radcliffe Hospital, University of Oxford, Oxford, United Kingdom; b Wellcome Centre for Integrative Neuroimaging, Department of Experimental Psychology, University of Oxford, Oxford, United Kingdom; c Cancer Research UK and Medical Research Council Oxford Institute for Radiation Oncology, Department of Oncology, University of Oxford, Oxford, United Kingdom; d Research Centre for Motor Control and Neuroplasticity, KU Leuven, Heverlee, Belgium; e Donders Institute for Brain, Cognition and Behaviour, Radboud University Nijmegen, Nijmegen, the Netherlands

**Keywords:** Diffusion-weighted MRI, Arcuate fasciculus, Neuroecology, Comparative neuroanatomy, Cortical tract representation

## Abstract

Evolutionary adaptations of the human brain are the basis for our unique abilities such as language. An expansion of the arcuate fasciculus (AF), the dorsal language tract, in the human lineage involving left lateralization is considered canonical, but this hypothesis has not been tested in relation to other architectural adaptations in the human brain. Using diffusion-weighted MRI, we examined AF in the human and macaque and quantified species differences in white matter architecture and surface representations. To compare surface results in the two species, we transformed macaque representations to human space using a landmark-based monkey-to-human cortical expansion model.

We found that the human dorsal AF, but not the ventral inferior fronto-occipital fasciculus (IFO), is left-lateralized. In the monkey AF is not lateralized. Moreover, compared to the macaque, human AF is relatively increased with respect to IFO. A comparison of human and transformed macaque surface representations suggests that cortical expansion alone cannot account for the species differences in the surface representation of AF.

Our results show that the human AF has undergone critical anatomical modifications in comparison with the macaque AF. More generally, this work demonstrates that studies on the human brain specializations underlying the language connectome can benefit from current methodological advances in comparative neuroanatomy.

## 1 Introduction

Language is the one behavior that is generally accepted to have some aspect unique to humans. This claim has been bolstered in the last decade by the discovery that the arcuate fasciculus (AF), a white matter tract traditionally implicated in language functions by connecting Broca's and Wernicke's areas (Geschwind, 1970), is dramatically expanded in the human brain compared to that of the macaque monkey and even the chimpanzee great ape (Rilling et al., 2008). Using diffusion-weighted magnetic resonance imaging (DW-MRI)-based tractography, Rilling et al. (2008) suggested that the human AF is unique in projecting to the middle and inferior temporal cortex. This finding has become the basis of a number of models of human language processing (Catani and Bambini, 2014, Roelofs, 2014).

Despite this initial success, a number of questions about the human AF remain. Most importantly, it remains unclear how the expanded AF fits with other findings of between-species differences in brain organization that have been proposed as crucial aspects of the human brain's unique architectural adaptations enabling language (Mars, Eichert, Jbabdi, Verhagen, & Rushworth, 2018). Left-hemispheric lateralization has traditionally been one of the hallmarks of the language network and might be related to a necessary increase in efficiency in larger brains (see Passingham, 2008 for a discussion). The cortical projections of the AF are thought to mimic this lateralization, but whether this generalizes to all aspects of the AF remains to be investigated. Other recent models highlight the contribution of ventral tracts in connecting language areas in the ventrolateral frontal and temporal and inferior parietal cortices (Makris & Pandya, 2009), although some authors emphasize the relative dominance of the AF (Rilling, Glasser, Jbabdi, Andersson, & Preuss, 2012). Another important new light on supposedly extended projections of the human AF is the increasing realization that expansion of the human brain has been remarkably non-uniform, resulting in selective increase in certain cortical territories and the displacement of homologous cortical areas between species (Mars et al., 2013, Van Essen and Dierker, 2007). Any claim of extension of a tract should thus be evaluated in the context of such cortical reorganization to rule out the possibility that what looks like a novel projection of a tract is simply a displacement or enlargement of a target area.

Since the original studies by Rilling et al., 2008, Rilling et al., 2012 identifying AF expansion in the human brain, there have to our knowledge been no studies comparing the AF between species using the same method. Advances in both the acquisition resolution of DW-MRI (McNab et al., 2009, Sotiropoulos et al., 2013) and analysis tools for comparative neuroscience (Mars et al., 2014) now allow a much more detailed quantification of the aspects of the AF that are different between the human brain and that of the macaque monkey model. Theoretical advances in our understanding of the differences in brain organization between species (Krubitzer, 2009, Mars et al., 2017), including those outlined above, also necessitate a reassessment of the unique aspects of the human AF. The goal of the present study, therefore, is to re-evaluate the extension of the human AF as compared to the macaque monkey using the same technique.

## 2 Materials and methods

We acquired DW-MRI data from humans and macaques for comparison of the AF using probabilistic tractography. Data were preprocessed using tools from FSL (www.fmrib.ox.ac.uk/fsl) and the in-house MR Comparative Anatomy Toolbox (Mr Cat, www.neuroecologylab.org).

### 2.1 Human data and preprocessing

Human *in vivo* DW-MRI data were provided by the Human Connectome Project (HCP), WU-Minn Consortium (Principal Investigators: David Van Essen and Kamil Ugurbil; 1U54MH091657) funded by the 16 NIH Institutes and Centers that support the NIH Blueprint for Neuroscience Research; and by the McDonnell Center for Systems Neuroscience at Washington University (Van Essen et al., 2013). The minimally preprocessed datasets of the first 25 subjects (15 female, age range 25–35 years, 22 right handed) of the Q2 release were used. Data acquisition and preprocessing methods are detailed in Uǧurbil et al., 2013, Sotiropoulos et al., 2013, and Glasser et al. (2013). In brief, 1.25 mm isotropic resolution diffusion-weighted data were collected across the entire brain on a customized 3T Siemens Skyra scanner using a monopolar Stejskal-Tanner diffusion encoding scheme (Stejskal & Tanner, 1965) with a slice-accelerated gradient echo EPI readout. Sampling in *q*-space included 3 shells at *b* = 1000, 2000, and 3000 sec/mm^2^. For each shell, 90 diffusion encoding gradient directions and 6 b = 0's were obtained twice, with the phase encoding direction reversed. Diffusion-weighted images were subsequently combined using the FSL TOPUP distortion correction tool (Andersson, Skare, & Ashburner, 2003) followed by eddy-current distortion and motion correction. T_1_-weighted images were acquired using an MPRAGE sequence at .7 mm isotropic resolution and aligned to the diffusion space as part of the HCP's minimum preprocessing pipeline (Glasser et al., 2013).

### 2.2 Macaque data

*Ex vivo* DW-MRI data were obtained from four rhesus monkeys (*Macaca mulatta*, 1 female, age at death range 4–14 years) using a 7T magnet with an Agilent DirectDrive™ (Agilent Technologies, Santa Clara, CA, USA). The brains were perfusion fixed and stored in formalin. Before scanning the brains were rehydrated in a phosphate-buffered saline solution and subsequently placed in fomblin for scanning purposes. Data were acquired using a 2D diffusion-weighted spin echo multi slice protocol with single line readout (DW-SEMS, TE/TR: 25 msec/10 sec; matrix size: 128 × 128; resolution .6 mm × .6 mm; number of slices: 128; slice thickness: .6 mm). In the monkeys, 9 non-diffusion-weighted (*b* = 0 sec/mm^2^) and 131 diffusion-weighted (*b* = 4000 sec/mm^2^) volumes were acquired with diffusion encoding directions evenly distributed over the whole sphere, except in one monkey were 7 non-diffusion-weighted images and 128 diffusion directions were collected.

Additionally, data from one male macaque (*Macaca fascicularis*) from a previous study (de Crespigny et al., 2005) were obtained and preprocessed as described in Jbabdi, Lehman, Haber, and Behrens (2013). Relevant imaging parameters for DW-MRI data were: 4.7T Oxford magnet equipped with BGA 12 gradients; 3D segmented spin-echo EPI 430 μm isotropic resolution, 8 shots, TE 33 msec, TR 350 msec, 120 isotropically distributed diffusion directions, *b* = 8000 sec/mm^2^.

RF bias field signal inhomogeneities were corrected in the average of the no-diffusion-weighted images in a robust iterative approach (FSL's FAST, Zhang, Brady, & Smith, 2001).

### 2.3 Tractography

Preprocessed diffusion-weighted images were subsequently processed using FMRIB's Diffusion Toolbox by fitting diffusion tensors (FSL's DTIFIT, Behrens et al., 2003) and by fitting a model of local fiber orientations including crossing fibers (FSL's BedpostX, Behrens, Berg, Jbabdi, Rushworth, & Woolrich, 2007). Up to three fiber orientations per voxel were allowed. Tractography was performed using FMRIB's Diffusion Toolbox, part of FSL. Registration warp-fields between each subject's space and standard space were created using FSL's FNIRT.

**Fig. 1:**
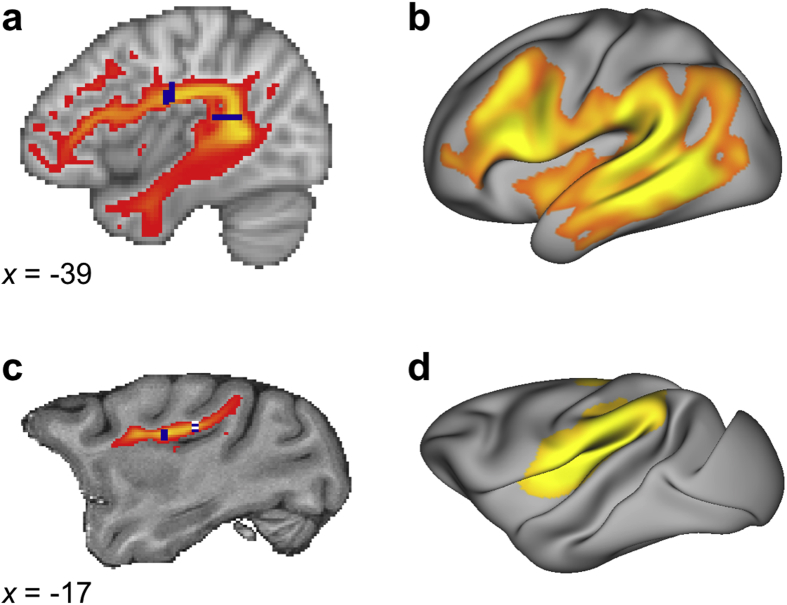
**Tractography results** (a) Tractogram of the left human AF projected on a standard brain (MNI). Shown is the result thresholded at .3 of the log-transformed and normalized group average (*n* = 25). Seed and target masks are shown in dark blue. (b) Surface representation of the human AF. Shown is the group average of smoothed representations thresholded at .9 of the log-transformed and normalized data (*n* = 25). (c) Tractogram of the average macaque AF projected on a standard brain (F99) (*n* = 5). Seed and target masks are shown in dark blue. The posterior mask is located at *x* = −13 and here projected to *x* = −17 for visualization. (d) Average surface representation of the macaque AF (*n* = 5) projected on a single individual macaque surface. The display parameters for the results in (c) and (d) are the same as in (a) and (b), respectively.

Masks for tractography were drawn in standard space (MNI152 for the human, F99 for the macaque (Van Essen, Glasser, Dierker, & Harwell, 2012)). The AF was defined as a tract running along the anterior-posterior dimension in frontal-parietal cortex along the fundus of the circular insular sulcus (Petrides, 2012). Seed masks were drawn just posterior to the level of the central sulcus (Fig. 1a, c). An axial target mask was placed in the parietal-temporal white matter posterior to the caudal end of the Sylvian fissure. Coronal exclusion mask through the subcortical white matter and extreme/external capsule just anterior to the anterior commissure and through subcortical white matter at the level of the seed prevented leaking into ventral longitudinal tracts. An additional exclusion mask of the mid–sagittal plane constrained the tracts to run within the ipsilateral hemisphere. The macaque AF is generally quite weak (Frey, Mackey, & Petrides, 2014) and therefore more difficult to reconstruct. The seed and targets were therefore placed in parts of the white matter where the principal fiber direction was most clear along the direction of the AF as defined in the atlas of Schmahmann and Pandya (2009). The seed was placed at the level of the central sulcus and the target mask was placed coronally at the level of the posterior thalamus. Similar exclusion masks as in the human were placed, and an additional exclusion mask through the superior parietal cortex at a location halfway through the upward spur of the cingulate sulcus was used to prevent fibers running dorsally. Tractography was performed by tracking from seed to target mask and also from target mask to seed mask. For the final tractography result both the regular and the inverse tractograms were averaged.

We compared the AF ‘dorsal language pathway’ to the 'ventral language pathway'. The ventral pathway is mostly formed by the fiber bundle running between the frontal and temporal cortices through the extreme capsule (Rilling et al., 2012) that is often termed the inferior fronto-occipital fasciculus (IFO) (Forkel et al., 2014) or extreme capsule fiber complex (Mars et al., 2016). To isolate this tract we followed a modified version of the approach proposed by Wakana et al. (2007). The IFO was defined as the tract running along the posterior-anterior dimension throughout the length of the temporal cortex to the frontal cortex. For the human, a coronal seed mask encompassing the ventral part of the hemisphere was placed just posterior of the most anterior part of the parietal-occipital sulcus (POS). A coronal target mask encompassing the entire hemisphere was placed just anterior to the genu of the corpus callosum. An exclusion mask consisted of the mid–sagittal plane and the coronal plane through the putamen except for the area where the IFO was expected. The macaque masks were placed in a similar location. Because the POS is oriented much more upright in the macaque, the coronal seed mask was placed between the splenium of the corpus callosum and the most anterior part of the POS.

These masks were warped to subject diffusion MRI space for tractography. Probabilistic tractography was performed using FSL's probtrackx2 using the same settings in both species: 5000 samples, 2000 steps, .5 mm step length, curvature threshold .2, randfib 1 with fibthresh .01. Individual subject tractograms (probabilistic streamline visitation maps, i.e., FSL's probtrackx2's fdt_paths) were warped back to standard space for group analyses. In order to account for the smaller voxel and brain size, we reduced the step length for macaque tractography to .25 mm.

Protocols and results files will be made available online upon acceptance of the paper (linked at www.neuroecologylab.org).

### 2.4 Lateralization index and dorsal/ventral ratio

The lateralization of each tract was calculated by using the tractograms of each tract of each subject separately for each hemisphere and applying the following formula:

$$lateralization=\frac{\sum_{v}right\_hemisphere\_tractogram-\sum_{v}left\_hemisphere\_tractogram}{\sum_{v}right\_hemisphere\_tractogram+\sum_{v}left\_hemisphere\_tractogram}$$

where *v* indicates all voxels or vertices. This results in a score between −1 (maximally left lateralized) and +1 (maximally right lateralized). Significance of the lateralization against zero was assessed using a two-sided Wilcoxon signed rank test. For all statistical tests we report *p*-values and Cohen's *d* as a measure of effect size.

Similarly, a comparison of the relative sizes of the dorsal and ventral language pathways (D/V index) was calculated as follows:

$$D/V\;index=\frac{\sum_{v}dorsal\_tractogram-\sum_{v}ventral\_tractogram}{\sum_{v}dorsal\_tractogram+\sum_{v}ventral\_tractogram}$$

were *v* indicates all voxels or vertices. This results in a score between −1 (maximally dominated by ventral pathway) and 1 (maximally dominated by dorsal pathway). This measure was calculated for each hemisphere separately and for the total tractograms summed across both hemispheres. We note that the absolute value of the D/V index is not the main concern of this analysis; the crucial result is in the comparison of the index across hemispheres and between species.

### 2.5 Surface analyses

To assess which part of the cortical grey matter might be reached by the AF, we created a surface representation of each human subject's AF tractogram using the approach described in Mars et al. (2018, bioRxiv). We calculated whole-brain vertex-wise connectivity matrices for a subset of four subjects, tracking from their individual mid-thickness surface to all voxels in the brain. Results of all subjects were subsequently averaged. A tract surface representation was then derived by multiplying its connectivity matrix with the average whole-brain tractogram.

Differences in surface representations between the left and right hemisphere were assessed on a vertex-by-vertex basis using permutation analysis as implemented in FSL's PALM (Winkler, Ridgway, Webster, Smith, & Nichols, 2014) on the smoothed tractogram representations (kernel of FWHM = 4, smoothing on the grey/white matter surface). We restricted the analysis to the surface area defined by the human average AF representation map (thresholded at .93 of the log-transformed and normalized representations). Results are thresholded at a statistical level of *p* < .05 corrected for multiple comparisons using family-wise error correction. Reported are effect sizes (Cohen's *d*) displayed onto an HCP average surface (Q1-Q6_R440).

Macaque AF surface representations we generated using the same multiplication method as described for humans. We computed an average whole-brain connectivity matrix of the five monkeys on the basis of a single individual macaque surface reconstruction. The surface and the macaque brains were warped to standard (F99) space to allow using the same surface modals in all five macaques. Macaque AF representations were derived from the averaged whole-brain connectivity matrix and smoothed (kernel of FWHM = 2 mm, smoothing on the individual monkey surface).

To derive D/V indices for surface data and surface lateralization of IFO, we also projected the IFO tractograms to the surface. Surface data visualization and manipulation were performed using Connectome Workbench (Marcus et al., 2011, www.humanconnectome.org).

### 2.6 Cortical expansion analysis

To assess whether cortical expansion between the human and macaque brain could account for the observed representation of AF, we applied a monkey-to-human cortical deformation procedure to the surface monkey data based on anatomical landmarks.

We mapped macaque tract representations to the F99 standard surface using Caret 5.65 (http://brainvis.wustl.edu/wiki/index.php/Caret:About). Then we applied a macaque-to-human transformation based on 23 landmarks developed by Van Essen and Dierker (2007) and previously used by Mantini, Corbetta, Romani, Orban, and Vanduffel (2013). Transformed macaque surface representations were then mapped to the 164k HCP mesh and resampled to 32k HCP space. To demonstrate the effect of cortical transformation, we applied the same procedure to a surface patch located at the border of TPO and TPT selected based on a macaque reference atlas (Lewis & Van Essen, 2000).

## 3 Results

We reconstructed AF in the human and macaque monkey brain using DW-MRI tractography. In both species, we used a frontally placed seed mask and a posteriorly placed target mask to reconstruct the pathways as they are known in the two species. We then used the resulting tractograms to compare a number of different measures describing their organization.

In the human, AF reached from the ventrolateral frontal cortex, via parietal cortex, all the way into the middle and inferior temporal lobe (Fig. 1). Although the body of a tract is the most reliable aspect of a tractography reconstruction, information where the tract reaches the grey matter is of most interest, since this shows which regions are connected by a tract. To establish which areas of the left hemisphere might be preferentially reached by the AF, we derived the surface representation of the tracts. Human AF representation dominated in the ventrolateral frontal cortex and the temporal lobe.

In contrast, the macaque AF was much weaker and terminated in the inferior parietal and posterior part of the temporal lobe. No invasion of the ventral part of the temporal lobe was observed. Macaque surface representations were most pronounced in inferior parietal lobe and in the insula. Representations in frontal areas in the macaque can be observed, but are relatively weak compared to posterior representations. The observed representations to the insula were likely to be driven by the close proximity of tractography seed and surface.

### 3.1 Lateralization

**Fig. 2:**
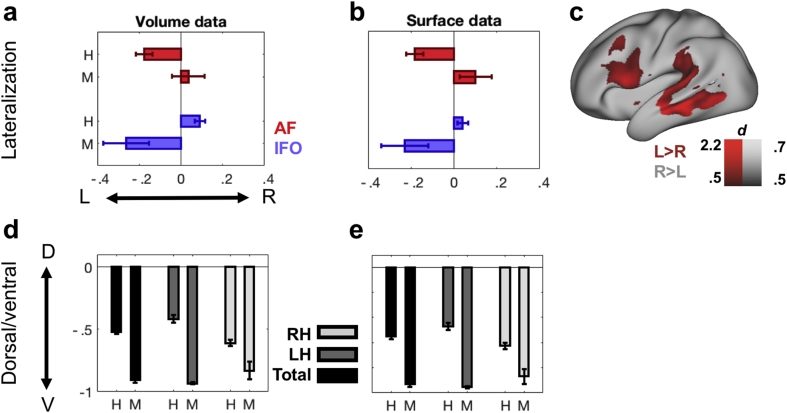
**Species differences** (a) Lateralization indices in human (H) and macaque (M) volume data for AF (red) and IFO (blue). (b) Lateralization indices in human and macaque surface data. (c) Interhemispheric differences of the human AF representations (Cohen's *d*) mapped onto the left hemisphere of a standard brain (Left-lateralization: red, right-lateralization: black). (d) Dorsal/ventral index in human and macaque volume data. (e) Dorsal/ventral index in human and macaque surface data.

In volume space, across the 25 human subjects tested AF was strongly left lateralized (Wilcoxon signed-ranks test *p* < .001, *d* = .67) (Fig. 2). For comparison, the IFO was right lateralized (*p* = .0080, *d* = .53). This pattern contrasts with that obtained in the four macaque subjects, who do not show a consistent left lateralization for the AF and a left rather than right lateralization for the IFO. Mimicking the results obtained in volume space, surface representations of the AF in the human showed significant left lateralization (*p* < .001, *d* = .69) and IFO showed a tendency towards right lateralization.

A vertex-by-vertex test on the human AF representations, testing whether AF representations are stronger on the left or right side showed a strong left lateralization in the frontal cortex around Broca's area (area 44/45) and in the vicinity of the inferior frontal junction area (Fig. 2c). Furthermore, left-lateralization was observed in the middle and inferior temporal cortex. A weaker right lateralization was found in a small part of the posterior part of the temporal cortex.

### 3.2 Relative increase of the dorsal pathway in the human brain

To assess whether the AF expanded in the human relatively to other pathways involved in language we calculated an index of the relative prominence of the dorsal AF and the ventral IFO in both species. As can be seen in Fig. 2, this ratio is extremely negative for the macaque, signifying the ventral IFO is much more pronounced relative to the dorsal AF, both when calculated for the tract volume as for the surface representation. For the human, this D/V index is much less negative in both cases, suggesting that the expansion of the language tracts is most prominently observed in AF.

The lateralization difference between human AF and IFO is also evident in the DV indices. The left and right D/V indices differ significantly in both volume space (*p* < .001, *d* = .77) and surface space (*p* < .001, *d* = .72).

### 3.3 Expansion or relocation of the human arcuate fasciculus

**Fig. 3:**
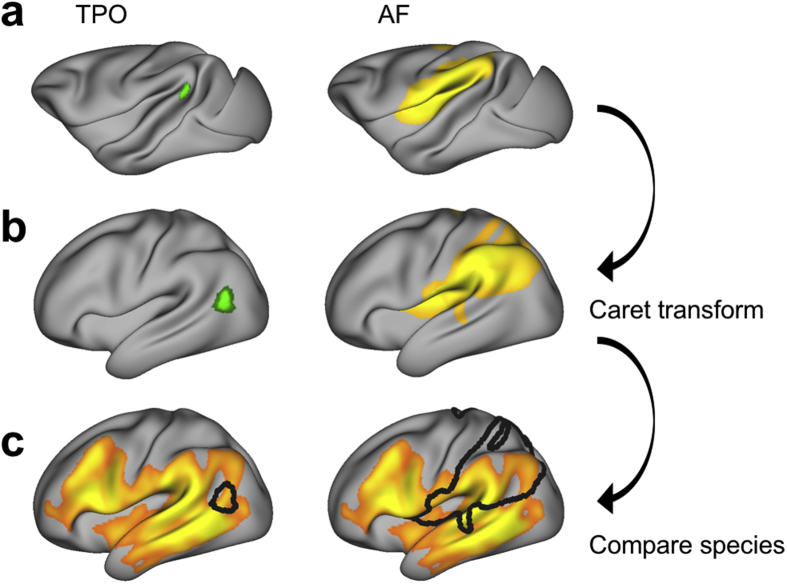
**Landmark-based transformation** (a) Regions of interest in the standard macaque brain surface (F99). Left panel: TPO selected based on macaque reference atlas. Right panel: Thresholded (.9) average surface representation of the left macaque AF, displayed as described in Fig. 1. (b) Regions of interest mapped onto the human standard brain surface by applying the Caret transformation (Van Essen & Dierker, 2007). (c) Species comparison can be performed with regions of interest in the same space. Shown is the thresholded (.9) average surface representation of the human AF, displayed as described in Fig. 1, overlaid with the macaque region of interest (black outline).

We assessed whether cortical expansion of the human compared to the macaque brain could account for our observed surface representation of the AF (Fig. 3). The landmark-based transformation of TPO from the macaque to the human cortical surface demonstrated that cortical expansion can drive relocation of surface regions between species. Before transformation, the TPO surface patch in the macaque brain was located within the caudal end of the superior temporal gyrus. The landmark-based transformation relocated TPO in the human brain to a position posterior and ventral with respect to the superior temporal sulcus.

Nonetheless, even when considering this transform, the macaque representation of AF in human space did not reach middle and inferior temporal areas. A direct comparison of human and macaque representation demonstrated that cortical expansion alone cannot account for the species differences in the surface representation of AF.

## 4 Discussion

It is almost canonical that the AF is one of the cortical innovations related to the uniquely human language ability, but the nature of its contribution is still a matter of debate. Indeed, many different specializations for the human AF have been suggested (Mars, Eichert, et al., 2018). Potential specializations that need to be considered are increased strength of AF and extension of the posterior representation, but also local cortical expansion resulting in relocation of representations and the modification of other language-related pathways. The present study sought to critically reassess some of these suggestions.

Human AF was left lateralized both in its body volume and in its cortical representation, but left lateralization of the latter was most obvious in ventrolateral frontal and temporal cortex. This pattern was markedly different to that of the ventral language pathway. As previously established (cf. Rilling et al., 2008), the AF invaded the middle and inferior temporal lobe in the human, but stayed dorsally in the macaque. The expansion of the AF in the human as compared to the macaque was evident by the ratio of brain volume of the AF and of the ventral IFO. In the macaque, this ratio was very negative, indicating the dominance of the ventral IFO over the dorsal AF, but this dominance is substantially reduced in the human. Finally, we aimed to test whether this increase in the AF pathway could be explained solely by the expansion of the human cortex, given that the human association cortex is much expanded compared to that of the macaque (Mars et al., 2017). Using the landmark-based Caret macaque-to-human transform, we showed that the cortical expansion differences between the two lineages cannot account for the differences we see in AF representation.

We have treated the AF here as a single fiber system, as is commonly done in the macaque tracer literature. Tractography is not limited to mono-synaptic pathways and thus best reconstructs fibers systems, rather than specific fibers. This leaves open the possibility of subdividing the results reported here into separate groups of connections. In the tracer literature, a similar approach of subdividing large fiber systems has been taken by Heilbronner and Haber (2014) for the cingulum bundle. Catani, Jones, and Ffytche (2005) have divided the AF into an anterior segment between Broca's territory in the frontal lobe and Geschwind's territory in the parietal cortex, a posterior segment between Geschwind's and Wernicke's territories, and a long segment spanning the entire range of the bundle. These different subdivisions showed diverging patterns of lateralization, with the long segment showing strong left lateralization, but the anterior segment between frontal and parietal cortex showing a weaker right lateralization (Thiebaut de Schotten et al., 2011). The current findings, demonstrating lateralization of representations in the ventral frontal cortex and temporal cortex, but some rightward lateralization in the posterior temporal cortex, can be interpreted as an extension of those results.

The so-called 'ventral language pathway' is formed by connections between the frontal and temporal cortex running through the extreme capsule. This contains at least two fiber bundles, the ventrally positioned uncinate fasciculus and the dorsally positioned IFO or extreme capsule fiber complex (e.g., Friederici & Gierhan, 2013). In addition, recent work has emphasized the role of fibers running longitudinally through the temporal lobe, such as the inferior and middle longitudinal fasciculus, in language (Catani and Bambini, 2014, Makris et al., 2013). To contrast the dorsal and ventral pathways, we have here pragmatically focused on the IFO as representative of the ventral pathway. This choice is justified as the inferior longitudinal fasciculus and middle longitudinal fasciculus do not project to the frontal lobe, and hence can thus be argued to not be part of a ventral frontotemporal pathway, and the uncinate is highly conserved between humans and macaques (De Schotten et al., 2011). It should be noted that the IFO differs in its posterior projections between human tractography studies and macaque tracer studies, but these effects are much smaller when using tractography in both species. Recent studies have shown posterior projections of the IFO in both macaques and marmosets when using tractography (Mars et al., 2016, Schaeffer et al., 2017, but see; Takemura et al., 2017).

The interpretation of the present tractograms is limited by the placement of tractography masks. Our tractography approach is aimed to be sensitive for species comparison, rather than for anatomical details within one species. The aim of the current project was thus not to establish the AF and the IFO per se, but to examine the various ways in which the known tract is special in the human brain. We have attempted to ensure that both acquisition and analysis of human and macaque data are comparable. Residual differences in data quality and resolution, however, are potential confounds of the tractography results. Nevertheless, given the markedly strong effects, we are confident that differing tracking performance only marginally affected the results.

In this study, we have investigated the cortical projections of the AF by means of surface representations of the tractography results. We acknowledge that problems with surface reconstructions, such as gyral bias, are well documented (e.g., Jbabdi and Johansen-Berg, 2011, Reveley et al., 2015), although more encouraging results have recently been reported (Donahue et al., 2016). Comfortingly, our lateralization results replicate across volume space, which looks at the body of the tract, and surface space, which indexes the grey matter terminations. Furthermore, our comparison of human AF representations as predicted by cortical expansion and reconstructed using tractography show a very different pattern across different cortical areas, much beyond the subtleties of differences in sulcal and gyral representations.

In sum, our results demonstrate that the human AF has expanded compared to the macaque, that this is accompanied by a left-lateralization of its frontal and temporal representations, and that it has expanded relative to other ventral tracts. This study confirms and extends previous comparative reports of the AF, using novel neuroanatomical approaches that allow a direct comparison of human and macaque data in a common framework. Future research on the anatomical basis of language will benefit from recent developments in comparative neuroimaging techniques to find shared and unique aspects of brain organization across species.

## Conflict of interest

The authors declare no competing financial interests.
